# Recent Advances and Developments in Mobile Health

**DOI:** 10.1155/2018/4747593

**Published:** 2018-12-30

**Authors:** Chase Q. Wu, Zongmin Wang, Guanling Chen, Denise Ferebee

**Affiliations:** ^1^New Jersey Institute of Technology, Newark, NJ, USA; ^2^Zhengzhou University, Zhengzhou, Henan, China; ^3^University of Massachusetts Lowell, Lowell, USA; ^4^LeMoyne-Owen College, Memphis, TN, USA

The traditional healthcare industry is undergoing a major paradigm shift due to the rapid advances and developments of mobile, wearable, and other wireless technologies. These Mobile Health (mHealth) technologies promise to bring tremendous benefits and opportunities to the diagnosis, prognosis, treatment, and prevention of human diseases for a better quality of life. In the meantime, mHealth also presents unprecedented performance and security challenges in the entire process of data collection, processing, analysis, synthesis, and visualization. For example, the advent of wearable technology has now made it possible to constantly monitor sophisticated biometrics for many people ranging from home athletes to chronic healthcare patients. A wide spectrum of devices are being designed as either a replacement of an existing healthcare monitor or a proposition for a new multifunction one. These devices typically require communication with a central healthcare system via cell phones or tablets, and thus, threats to the data at rest and in transit still exist, apart from a potential risk of misuse via patient profiling. Therefore, it is crucial to design and implement new mHealth technologies to build reliable, accurate, efficient, and secure healthcare environments for optimal patient care. The wide deployments of such wearable monitoring devices have also raised a critical issue to health informatics caused by the sheer volume and high complexity of health data collected anywhere and anytime. Machine learning and big data-oriented algorithms, models, systems, and platforms are needed to support the analysis, use, interpretation, and integration of diverse health data.

This special issue includes 14 research articles, addressing various aspects of the recent mHealth advances and developments that use mobile and wireless devices to improve healthcare outcomes, services, and research. In the article titled “Mobile Aid to Assist with Care Decisions in Children with Autism Spectrum Disorder (ASD),” A. Khan et al. developed an autism spectrum disorder intervention application that provides an outlet for children to express their emotions while providing an uncomplicated environment. In the article titled “Systems and WBANs for Controlling Obesity,” M. S. Mohammed et al. explored the use of wireless body area networks (WBANs) and related systems for controlling obesity and proposed to integrate such technologies into an intelligent architecture. In the article titled “Indication of Mental Health from Fingertip Pulse Waves and Its Application,” M. Oyama-Higa and F. Ou explored the use of the largest Lyapunov exponent (LLE) of the attractor to provide an effective indicator of mental health. In the article titled “A New Remote Health-Care System Based on Moving Robot Intended for the Elderly at Home,” B. Zhou et al. developed specialized robotics technologies for remote geriatric care. In the article titled “A Mobile Multimedia Reminiscence Therapy Application to Reduce Behavioral and Psychological Symptoms in Persons with Alzheimer's,” D. Imtiaz et al. developed a mobile technology-based solution to address behavioral and psychological symptoms of dementia (BPSD) that occur in individuals with Alzheimer's dementia. In the article titled “QRS Detection Based on Improved Adaptive Threshold,” X. Lu et al. designed an adaptive threshold algorithm for QRS detection that can be used in mobile devices. In the article titled “Genuine and Secure Identity-based Public Audit for the Stored Data in Healthcare Cloud,” J. Zhang et al. constructed an identity-based data-auditing system where an algorithm is used to calculate an authentication signature. In the article titled “Modeling Medical Services with Mobile Health Applications,” Z. Wang et al. designed a medical service equilibrium model for evaluating the influence of mHealth applications on the medical service market to balance the supply of doctors and the demand of patients. In the article titled “Chinese Mobile Health APPs for Hypertension Management: A Systematic Evaluation of Usefulness,” J. Liang et al. conducted a study of Chinese mobile health APPs for hypertension management to investigate the difference and effectiveness of the APPs between mainland China and other places. In the article titled “Leveraging Multiactions to Improve Medical Personalized Ranking for Collaborative Filtering,” S. Gao et al. constructed a medical Bayesian personalized ranking (MBPR) over multiple users' actions based on a simple observation that users tend to assign higher ranks to healthcare services that are meanwhile preferred in users' other actions. In the article titled “A Systematic Review on Recent Advances in mHealth Systems: Deployment Architecture for Emergency Response,” E. Gonzalez et al. conducted a broad survey of recent advances in mHealth systems. In the article titled “An Ensemble Multilabel Classification for Disease Risk Prediction,” R. Li et al. explored the use of ensemble multilabel classification for disease risk prediction. In the article titled “Semiautomatic Segmentation of Glioma on Mobile Devices,” Y.-P. Wu et al. studied hard edge multiplicative intrinsic component optimization to preprocess glioma medical images. In the article titled “Handling Data Skew in MapReduce Cluster by Using Partition Tuning,” Y. Gao et al. explored the use of partition tuning-based skew handling (PTSH) to make improvements over the traditional MapReduce model in processing large healthcare datasets.

